# Contraceptive use and determinants among sexually active unmarried adolescents and young women (aged 15–24 years) in East Africa: insights from Demographic and Health Survey data

**DOI:** 10.1093/inthealth/ihae079

**Published:** 2024-11-08

**Authors:** Saba Hailu, Helina Heluf, Galana Mamo Ayana, Belay Negash

**Affiliations:** School of Public Health, College of Health and Medical Science, Haramaya University, Ethiopia, P.O. BOX: 235; School of Nursing and Midwifery, College of Health and Medical Science, Haramaya University, Ethiopia, P.O. BOX: 235; School of Public Health, College of Health and Medical Science, Haramaya University, Ethiopia, P.O. BOX: 235; School of Public Health, College of Health and Medical Science, Haramaya University, Ethiopia, P.O. BOX: 235

**Keywords:** contraceptive use, DHS, East Africa, sexually active, unmarried adolescents, unmarried young women

## Abstract

**Background:**

Low contraception usage among adolescents is a significant public health issue, leading to a rise in unintended pregnancies and adolescent childbearing in sub-Saharan Africa. Despite global efforts to improve access to contraception, sexually active adolescents and young women in East Africa are often overlooked, exposing them to substantial health risks. This study explored the factors influencing contraceptive use among unmarried and sexually active adolescents and young women in East African countries.

**Methods:**

National representative data from the 2016 Demographic and Health Survey for eight East African countries were used in the analysis. Data processing and analysis were performed using STATA 17 software. A multilevel mixed-effect logistic regression was used to identify determinants of contraceptive use at p<0.05.

**Results:**

A total of 7813 sexually active unmarried adolescents and young women were considered for the final analysis. Among these young women, 24.9% were using a contraceptive method. Age, place of residence, knowledge of contraceptive methods, employment status and educational attainment were identified as significant determining factors of contraceptive use among sexually active unmarried adolescents and young women (aged 15–24 y) in East Africa.

**Conclusions:**

Contraceptive utilization among sexually active unmarried adolescents and young women was relatively low in East African countries. Addressing the existing inequalities in access to contraceptive services for rural adolescents and young women in East African countries is crucial. Prioritizing policies that implement comprehensive sexual education is essential to enhance their knowledge of contraceptives and empower them to make informed decisions.

## Introduction

Today, there are 1.2 billion youth aged 15–24 y globally, accounting for one out of six people worldwide. In Africa, the number of youth is growing, with an absolute count nearing 226 million, accounting for 19% of the global youth population, making them the largest generation the region has ever had to raise.^1^ Young people undergo a transition from childhood to adulthood and enter their reproductive years.^2^ Youth is a time of both risk and opportunity, and their sexual behaviors and decisions impact their future social, educational and economic prospects.^3,4^

Adolescent pregnancies are a global problem, occurring in high-, middle- and low-income countries. An estimated 17 million adolescent girls give birth every year, and most of these births occur in low- and middle-income countries (LMICs),^5^ accounting for 16% of all births in sub-Saharan Africa (SSA).^2^ Trend analysis reports comparing adolescent sexual and reproductive health (SRH) indicators from 1995 to 2000 with those from 2015 to 2020 indicate that the adolescent birth rate has declined from 60 to 43 births per 1000 girls aged 15–19 y. However, the global rate has masked sharp differences in levels and trends across regions.^6^ Studies state that adolescent pregnancy remains a common health problem in East Africa.

In East African countries, adolescents' most recent births and pregnancies were unintended, ranging from 41.3% in Tanzania to 61.7% in Kenya.^7^ A high prevalence of unintended pregnancy has been reported among sexually active unmarried adolescents and young women.^8–10^ A study in Rwanda indicated that the majority of women who underwent unsafe abortions were unmarried and aged <25 y.^11^ Ensuring contraceptive accessibility and availability to all reproductive aged women is an essential step to eliminate unintended pregnancy.^12^ Meeting the need for modern contraception among adolescents would avert 2 million unplanned births and 3.2 million abortions among adolescent girls in LMICs.^13^ High fertility, coupled with declining mortality, has led to high population growth in Africa.^14^ One study highlighted that meeting the contraceptive demand for unmarried adolescents has a higher impact on reducing fertility in SSA.^15^

Young women, especially adolescents, are at a higher risk of mortality associated with pregnancy and childbirth. It is estimated that one-quarter of 20 million unsafe abortions and 70 000 abortion-related deaths are attributed to adolescent girls. They are twice as likely to die during childbirth than women aged in their 20s.^16^ The risk of death among women aged 15–24 y is higher because most of the pregnancies in this specific age group are unplanned.^17^

The use of modern contraceptives varies by age and marital status. Unmarried young people face challenges in using SRH services in settings where premarital sexual activity is stigmatized.^18,19^ In Africa, a high proportion of people become sexually active during adolescence. For instance, the median age at first sexual intercourse for unmarried young women aged 15–24 y in Tanzania, Uganda, Zambia and Malawi is 16 y; in Rwanda and Ethiopia, the median age of sexual imitation is 17 y.^20^ Teenage pregnancy in eastern and South African countries is high, with an estimated 25% of young women giving birth before the age of 18 y.^21^ Several studies have highlighted that young women face obstacles to obtaining contraceptive information and services from health facilities because providers tend to be judgmental and scold sexually active unmarried girls when they present themselves for services.^6,22^

Existing research on the use of contraception among young women is well documented,^23,24^ however, less is known about contraceptive use among sexually active unmarried adolescents and young women, and it has not incorporated East African countries. Available evidence showed that sexually active unmarried adolescents generally do not intend to become pregnant, while married adolescents either wish to avoid pregnancy at a young age or prefer to delay a second pregnancy.^15^ However, only 40% of adolescents in LMICs use an effective contraceptive method.^10,21^ Despite clear needs, many adolescents struggle to access contraception, leading to unplanned pregnancies even when contraceptive intentions are strong. There are significant disparities in contraceptive use across age groups, and young people often lack sufficient access to SRH information and services.^25^

The SRH needs of unmarried sexually active young women remains an under-researched area given that premarital sex is not culturally acceptable in many parts of African countries. Meeting the contraceptive needs of young women is an essential component of strategies to enhance their well-being. Indeed, findings from this study could provide an important insight into the intervention areas of this important population for programmers and policymakers. Therefore, the current study aimed to identify the determinants of contraceptive use among sexually active unmarried adolescents and young women (aged 15–24 y) in East Africa.

## Materials and methods

### Study setting and data source

The East Africa region comprises 19 countries, namely, Burundi, Comoros, Djibouti, Ethiopia, Eritrea, Kenya, Madagascar, Malawi, Mauritius, Mozambique, Reunion, Rwanda, Seychelles, Somalia, Somaliland, Tanzania, Uganda, Zambia and Zimbabwe. This study utilizes secondary data from the Demographic and Health Survey (DHS). Among these, 13 countries have DHS data available, while six—Djibouti, Somalia, Somaliland, Seychelles, Mauritius and Reunion—do not. Of the 13 countries with DHS data, eight—Burundi, Ethiopia, Malawi, Rwanda, Tanzania, Uganda, Zambia and Zimbabwe—have data from 2015–2016, which include relevant and current variables. DHS data from the remaining countries (Eritrea and Madagascar) are from before 2010 (Eritrea: 2002; Madagascar: 2008). Thus, this study focuses on the eight countries with 2015–2016 DHS data to ensure that the analysis is based on the most recent and relevant information.

The Demographic and Health Survey (DHS) is a nationally representative, cross-sectional surveys that collect data on essential health indicators, including mortality, morbidity, family planning, fertility, and maternal and child health. The DHS uses a two-stage stratified sampling technique to select study participants. The DHS survey used a two-stage stratified sampling method. During stage one, regions, provinces and states are stratified into urban and rural areas. Then clusters from each region are sampled, proportional to size. In sampled households, all women aged 15–49 y who consent to participate in the survey are interviewed. There was a total of 11 588 youths in the 5 y preceding each country's DHS. Of those, 7813 youths fully fulfilled the inclusion criteria for the current study. All unmarried adolescent girls aged 15–19 y, unmarried young women aged 20–24 y, and those who ever had sex (a total weighted sample of 7813), were considered for the final analysis. Detailed information on the survey country, the number of adolescents in each country, eligibility and the actual number of women for each country is provided in Table 1.

**Table 1.** Sociodemographic characteristics of sexually active unmarried adolescents and young women (aged 15–24 y) in East Africa

Variable	Weighted frequency (%)	Percentage
Age, y		
15–19	3855	49.3
20–24	3958	50.7
Residence		
Urban	3247	41.6
Rural	4566	58.4
Educational level		
No formal education	188	2.41
Primary	2877	36.8
Secondary	4170	53.4
College and above	578	7.4
Wealth quintile		
Lowest	873	11.2
Second	1039	13.3
Middle	1257	16.1
Fourth	1718	21.9
Highest	2926	37.5
Country		
Burundi	572	7.3
Ethiopia	272	3.5
Malawi	1610	20.6
Rwanda	960	12.3
Tanzania	953	12.2
Uganda	1351	17.3
Zambia	1582	20.3
Zimbabwe	513	6.6
Current employment status		
Unemployed	4083	52.3
Employed	3729	47.7

The data for these eight East African countries were obtained from the official database of the DHS program (www.measuredhs.com) after authorization was granted via an online request explaining the purpose of our study. Important variables were extracted as dependent and independent from the individual record (IR) file dataset. The IR file is a comprehensive dataset containing information on individual women who participated in the survey. This file provides detailed demographic, health and reproductive information, including socioeconomic background, reproductive health status and marriage and sexual activity. The DHS program collects contraceptive use through a single dichotomous (Yes/No) question, which is used in all countries around the world.

### Variables

#### Outcome variable

The dependent variable was current contraceptive use at the time of the survey. Contraceptive use is assessed by categorizing young women who responded that they or their partner(s) were using any contraceptive methods (modern and traditional methods) to avoid or delay pregnancy at the time of the survey. Modern contraceptive methods such as male sterilization, female sterilization, pills, injectable, intrauterine device, implant, male condom, female condom, emergency contraceptive and lactational amenorrhea and traditional methods (the rhythm and withdrawal method) were assessed among sexually active young women. The response was dichotomized into two categories indicating users and non-users of contraceptive methods.

The contraceptive behavior of young women was assessed based on their sexual activity. Young women who reported having sexual intercourse 4 wk before the survey were regarded as being sexually active. Non-sexually active and pregnant adolescents and young women were excluded from the analysis.

#### Independent variables

The following variables were considered as potential predictors of current contraceptive use: age, place of residence, household-level wealth quintile (Lowest, Second, Middle, Fourth and Highest), educational status, employment status, age at first sexual intercourse, history of abortion and childbirth. Knowledge of contraceptives was also treated as one of the predictor variables: it was defined as knowing at least one contraceptive method (either modern or traditional). A dichotomous variable was created, with knowing contraceptive methods defined as knowing at least one type of contraceptive method (either modern or traditional), while having no contraceptive knowledge was defined as the young woman being unaware of any contraceptive methods. The other variables included being exposed to contraceptive messages via television, radio or newspapers in the last few months before the survey.

### Statistical analysis

Data processing and analysis were performed using STATA 17 software (StataCorp LLC, College Station, TX, USA). Before conducting any statistical analysis, the data were weighted using sampling weights, primary sampling units (clusters) and sample domains (strata) to ensure that the data were representative and to instruct STATA to account for the sampling design when calculating standard errors to obtain accurate statistical estimates.

The important assumptions such as χ^2^ and multicollinearity assumptions were checked prior to fitting the statistical model. The assumptions of observation independence and equal variance across clusters were violated. Therefore, to ensure the reliability of SE and unbiased estimate, multilevel advanced statistical modeling was important to overcome the violated independence assumption and to consider the variability between clusters.

The multicollinearity assumption was checked using the variance inflation factor (VIF), where the VIF measures the amount of multicollinearity in a set of multiple regression variables. The VIF of the predictor variables was <3, indicating there was no multicollinearity between variables. Linearity in the Logit assumption was checked using the Box-Tidwell variables transformation, and we have confirmed that linearity in the logit of contraceptives was fulfilled.

The bivariable multilevel mixed-effect logistic regression model was fitted for all independent variables, and those variables with p=0.20 in the bivariable multilevel mixed-effects logistic regression analysis were candidates for multivariable two-stage mixed-effects logistic regression. A mixed-effect model with the lowest Akaike information criteria (AIC) and Bayesian information criteria (BIC) was selected. Significant determinants of contraceptive use were declared significant determinants of contraceptive use with p=0.05. An OR with a 95% CI was used to measure the strength of the association. The fixed-effects submodel was used to estimate the association between contraceptive use and explanatory variables. To measure cluster variation, the intracluster correlation coefficient (ICC) SD was used. Besides, the model comparison was performed based on AIC and BIC.

## Results

### Sociodemographic characteristics of the study participants

The ages of the young women who participated were fairly distributed between 15–19 and 20–24 y, with 49.3% and 50.6%, respectively. More than one-half of the study participants (58.4%) lived in rural areas. Approximately one-fifth of respondents were from Malawi (20.6%) and about one-fifth from Zambia (20.2%). Of all the participants, more than one-half (53.4%) had a secondary level of education and 2926 young women (37.5%) were in the highest wealth quintile. Regarding sexual debut, 86% of young women experienced sexual intercourse aged 15–19 y. Nearly one-half of women (47%) were not employed at the time of the survey (Table 1).

### Use of contraceptive methods and information

Among 7813 sexually active unmarried adolescents and young women aged 15–24 y, 1942 (24.9%) were using any type of contraceptive method (92.6% modern methods and 7.4% traditional methods). Among modern contraceptive methods, the male condom was the most frequently used (39.9%), while only one participant's partner used male sterilization. Almost all young women (99.1%) had knowledge of contraceptive methods (Table 2).

**Table 2.** Knowledge, information and use of contraceptives among sexually active unmarried adolescents and young women (aged 15–24 y) in East Africa

Variable	Weighted frequency	Percentage
Knowledge of contraceptive methods		
Yes	7745	99.1
No	68	0.9
Currently use contraceptive method		
Yes	1942	24.9
No	5871	75.1
Type of contraceptive method currently using		
Pills	107	5.5
IUCD	8	0.4
Injections	509	26.2
Male condom	774	39.9
Periodic abstinence	100	5.2
Withdrawal	40	2.1
Other traditional	4	0.2
Implants	364	18.7
Lactational amenorrhea	6	0.3
Female condom	10	0.5
Emergency contraception	14	0.7
Standard days method	5	0.3
Heard about FP on TV		
Yes	1834	23.5
No	5979	76.5
Heard FP on radio last few months		
Yes	3489	44.7
No	4324	55.3
Heard FP in newspaper/magazine		
Yes	1337	17.1
No	6476	82.9
Heard FP by text messages on mobile/phone		
Yes	431	5.5
No	7382	94.5
Age at first sex, y		
15–19	6778	86.8
20–24	1034	13.2
Ever terminated pregnancy		
Yes	204	2.6
No	7609	97.4
Live children		
Yes	2482	31.8
No	5331	68.2

FP: family planning; IUCD: intrauterine contraceptive device.

### Determinants of contraceptive use among sexually active unmarried adolescents and young women in eight East African countries

#### Random effects

The results of the null model revealed that there was statistically significant variability in the odds of contraceptive use, with a community variance of 0.32. The ICC in the null model was 0.96, which indicates that about 9.6% of the total variation in contraception use was attributed to the difference between clusters; the remaining 90.4% of the total variability in contraceptive use among unmarried adolescents and young women was attributed to individual variability.

#### Fixed effects

In the bivariable logistic regression model, age, place of residence, educational attainment, wealth index, knowledge of contraceptive methods, respondents' current employment status and hearing about contraceptive methods from TV, radio or by reading newspapers were significantly associated with contraceptive use. However, in the final multivariable multilevel logistics model, age group, residence, knowledge of contraceptive methods, respondents' current employment status and educational attainment were significant determinants of contraceptive use among sexually active unmarried youth (aged 15–24 y) (Table 3).

**Table 3.** Factors associated with contraceptive use among sexually active unmarried adolescents and young women (aged 15–24 y) in East African countries

	Models			
Variable	Null model AOR (95% CI)	Model I AOR (95% CI)	Model II AOR (95% CI)	Model III AOR (95% CI)
**Current age, y**				
15–19		Ref.		Ref.
20–24		2.30 (1.22 to 2.57)		2.37 (1.22 to 2.54)*
**Educational status**				
No formal education		Ref.		Ref.
Primary		1.32 (0.88 to 1.98)		1.29 (0.86 to 1.95)
Secondary		1.50 (0.99 to 2.25)		1.50 (0.99 to 2.27)
Higher		2.59 (1.01 to 3.49)		2.62 (1.03 to 3.55)*
**Wealth quintile**				
Lowest		Ref.		Ref.
Second		0.96 (0.76 to 1.20)		0.96 (0.77 to 1.21)
Middle		0.97 (0.78 to 1.20)		0.96 (0.77 to 1.19)
Fourth		1.02 (0.83 to 1.25)		0.94 (0.76 to 1.17)
Highest		0.94 (0.77 to 1.16)		0.84 (0.66 to 1.05)
**Current working status**				
No		Ref.		Ref.
Yes		1.18 (1.06 to 1.32)		1.15 (1.03 to 1.28)*
**Knowledge of contraceptive methods**				
No		Ref.		Ref.
Yes		2.37 (1.73 to 3.25)		2.38 (1.74 to 3.26)*
**Heard about contraceptives from radio**				
No		Ref.		Ref.
Yes		0.96 (0.85 to 1.08)		0.96 (0.85 to 1.09)
**Heard about contraceptives from TV**				
No		Ref.		Ref.
Yes		1.10 (0.95 to 1.28)		0.99 (0.85 to 1.15)
**Read about contraceptives from newspaper**				
No		Ref.		Ref.
Yes		1.53 (1.32 to 1.77)		1.40 (1.20 to 1.63)*
**Place of residence**				
Rural			Ref.	Ref.
Urban			2.27 (1.14 to 2.42)	2.21 (1.05 to 2.40)*
**Country**				
Burundi			Ref.	Ref.
Ethiopia			1.30 (0.92 to 1.83)	1.26 (0.88 to 1.80)
Malawi			1.05 (0.83 to 1.34)	1.07 (0.84 to 1.37)
Rwanda			1.24 (0.96 to 1.60)	1.16 (0.90 to 1.50)
Tanzania			1.68 (1.31 to 2.15)	1.55 (1.19 to 2.02)*
Uganda			1.23 (0.97 to 1.56)	1.18 (0.92 to 1.52)
Zambia			0.90 (0.71 to 1.15)	0.91 (0.71 to 1.17)
Zimbabwe			1.91 (1.45 to 2.52)	1.74 (1.30 to 2.32)*
**Random effects**				
Community variance	0.31	0.08	0.09	0.08
ICC (%)	28.76	25.20	27.34	24.06
**Model comparison**				
AIC	8752.38	8594.668	8672.706	8557.28
BIC	8766.308	8699.119	8742.342	8717.439

AIC: Akaike information criteria; AOR: adjusted OR; BIC: Bayesian information criteria; ICC: intracluster correlation coefficient. *p value <0.05.

The final logistic regression model showed that the odds of contraceptive use was 2.37 (adjusted OR [AOR] 2.37, 95% CI 1.22 to 2.54) times higher among the 20–24 y age group compared with the 15–19 y sexually active unmarried age group. The likelihood of contraceptive use was 2.21 (AOR 1.21, 95% CI 1.05 to 2.40) times higher among urban compared with rural dwellers. In addition, having knowledge of contraceptive methods increases the odds of contraceptive use by 2.38 (AOR 2.38, 95% CI 1.74 to 3.26) times compared with their counterparts. Participants who obtained information about contraceptive methods from newspapers were 40% (AOR 1.40, 95% CI 1.20 to 1.63) more likely to use contraceptives compared with those who did not read a newspaper. Moreover, the likelihood of contraceptive use was 1.15 (AOR 1.15, 95% CI 1.03 to 1.28) times more likely among employed study participants compared with those who were unemployed. The current study revealed that the odds of contraceptive use were 2.62 (AOR 1.62, 95% CI 1.03 to 3.55) times higher among participants with college educational attainment compared with participants who had no formal education. Finally, the results from the final multilevel logistic regression model indicated that the likelihood of using a contraceptive method was 1.55 (AOR 1.55, 95% CI 1.19 to 2.02) and 1.74 (AOR 1.74, 95% CI 1.30 to 2.32) times higher among participants from Tanzania and Zimbabwe, respectively, compared with those from Burundi.

## Discussion

This article examined factors affecting contraceptive use among sexually active unmarried young women in East African countries. This study found that 24.8% of sexually active unmarried young women aged 15–24 y currently used any type of contraceptive method. Age, place of residence, knowledge of contraceptive methods, employment status and educational attainment were identified as significant determining factors of contraceptive use among sexually active unmarried youth (aged 15–24 y) in East Africa.

The prevalence of contraceptive use in our study is higher compared with studies conducted in Nigeria (15.8%)^26^ and Rwanda (17.4%).^27^ This finding could perhaps be explained by the difference in the type of contraceptive methods included in the studies. Our study has incorporated both traditional and modern methods; however, the aforementioned studies excluded traditional methods from their analyses. There is a greater possibility that unmarried young women would use traditional methods because sexual activity is only considered acceptable within marriage in some settings. Hence, excluding traditional methods might have underestimated the prevalence of contraceptive use. We found considerable variation in contraceptive use between a study conducted in Ghana (43%)^28^ and one performed in SSA countries (38.6%).^29^ This might be due to supportive social networks, respecting confidentiality and increased accessibility and affordability of contraceptive methods, as they were cited as motivators of contraceptive use among young people in SSA countries.^30^

In this study, age is identified as a predictor of contraceptive use among sexually active unmarried women. This finding is comparable with that from studies conducted in Nigeria^26^ and Ethiopia.^31^ This is expected because young women aged 20–24 y are mature and assumed to have a better understanding of the consequences of engaging in sexual activity without contraception than adolescents.^32^ Literature has reported that adolescents face barriers that might inhibit them from accessing contraceptive methods, such as fear of side effects, cost, misconceptions and lack of knowledge.^33,34^ On the other hand, adolescents are traditionally financially dependent on their parents, and obtaining money for contraceptives can be difficult. Cultural norms of abstinence from premarital sex, which is related to the preservation of family honor coupled with the cost of contraceptives, leads to unmet needs. Removing financial barriers through subsidized contraceptive services and educational programs is crucial for empowering adolescents to make informed choices. Engaging in sexual activity without marriage is considered a promiscuous life in most parts of Africa and sexually active adolescents might not be confident enough to access methods, fearing the feedback from providers, as well as the community.^35^

In this study, the likelihood of contraceptive use by young women was 2.21 times higher among participants from urban residences compared with those from rural residences, which is consistent with findings from studies conducted in South Western Nigeria^36^ and India.^37^ This is not surprising given that significant urban–rural contraceptive service inequalities have been documented in accessing contraceptives among adolescents in SSA countries.^38^ Alleviating service barriers significantly improves access for adolescents and youth, leading to a reduction in unmet needs for these age groups.^39^ This has positive implications for both individual and public health outcomes. Moreover, being sexually active is a taboo for unmarried adolescents and young women, especially in rural areas, which might limit their ability to access contraceptives, even if they are available.^35^ This highlights the need to expand youth-friendly clinics in rural areas and the importance of implementing school-based awareness programs and incorporating SRH education into the curriculum in rural settings.

Almost all the adolescents and young women (99%) in this study were aware of contraceptive methods. Our results provide evidence that young women who are knowledgeable about contraceptive methods are twofold more likely to use contraceptives compared with young women who lack knowledge. In line with our findings, studies performed among young girls in Tanzanian and Malawi^40,41^ share similar findings. This is probably because the majority of the participants in this study had above primary (36.8%) and secondary (53.4%) school education. This may be related to understanding the health risks of facing pregnancy at an early age, and knowing the consequences helps them with informed decision-making.^42^ This might have ultimately increased their demand for contraceptive use.

This study, in agreement with studies conducted in Ethiopia,^43^ Libiya^44^ and SSA,^45^ elucidates that contraceptive use is highest among employed adolescents and young women compared with unemployed young women. One possible explanation for this finding is that employed women have their own source of income and autonomy and therefore are presumably not under any financial constraints to access contraceptive-related services. Another study also emphasized that because female employees are financially empowered they have an opportunity to address issues presented by sociocultural and health system factors.^46^ To maximize contraceptive use among adolescents and young women in East African countries, it is essential to consider the affordability of contraceptive methods.

The findings from the current study indicate that participants who obtained information about contraceptive methods from a newspaper were 1.35 times more likely to use contraceptives compared with those who did not read a newspaper. This result is supported by studies conducted in Ghana^47^ and North Western Tanzania.^40^ This implies that gaining access to contraceptive messages might be an effective way of developing a positive attitude and behavior towards the use of contraceptives, which in turn leads to improved use of contraception.^48^

Finally, the current study revealed that participants with secondary and postsecondary educational attainment were 2.61 more likely to use contraceptives compared with those with no formal education. This is consistent with studies conducted in Ghana and Zambia.^49,50^ Attending formal education provides the opportunity to be exposed to information related to SRH, including the importance of modern contraceptive use, which clarifies misconceptions held against modern contraceptive utilization.^51,52^ Moreover, educated girls are more likely to recognize the benefits of using contraceptives in their lives. They may also have career ambitions and therefore wish to postpone having children.^25^

## Strengths and limitations

The results from this study are from datasets based on weighted nationally representative data covering eight countries in East Africa. These data are generalizable to sexually active unmarried adolescents and young women residing in those countries. However, despite the current study's strengths, it has certain limitations. For example, the cross-sectional nature of the study design could have influenced the strength of any conclusions drawn from the results regarding causation or relationships. In addition, the study depends on self-reported information, which might be subject to recall bias, as well as social desirability bias, which may have resulted in the prevalence of the outcome variable being underestimated.

## Conclusions

Nearly one-quarter of young sexually active young women were using contraceptive methods. Age, place of residence and knowledge of contraceptive methods, respondents’ current employment status and educational attainment were significantly associated with contraceptive use. Addressing the existing inequalities in access to contraceptive services for rural adolescents and young women in East African countries is crucial. Prioritizing policies that implement comprehensive sexual education is essential to enhance their knowledge of contraceptives and empower them to make informed decisions. Furthermore, improving access to education and employment opportunities for girls and young women in these regions is critical. Laws and policies must specifically address the needs and priorities of unmarried adolescents and young women, ensuring equitable access to contraceptives regardless of age, marital status or existing inequalities.

## Data Availability

The East Africa Demographic and Health Surveillance datasets are open and available online, so they can be accessed from the Measure DHS website www.measuredhs.com) via an online request by explaining the objective of the study. The datasets analyzed during the current study are available from the corresponding author upon reasonable request.
